# Transformational leadership and employability among support staff workers with long tenure

**DOI:** 10.3233/WOR-230371

**Published:** 2024-07-03

**Authors:** Maike Blumenthal, Beatrice I.J.M. Van der Heijden, Rikkie L. Dautzenberg, Cécile R.L. Boot

**Affiliations:** a Radboud University, Nijmegen, The Netherlands; b Institute for Management Research, Radboud University, Nijmegen, The Netherlands; c Faculty of Management, Open Universiteit, Heerlen, The Netherlands; d Department of Marketing, Innovation and Organisation, Ghent University, Ghent, Belgium; e School of Business Hubei University, Wuhan, China; f Kingston Business School, Kingston University, London, UK; g Reflect Forward, Organizational Consultancy, Nijmegen, The Netherlands; h Behavioural Science Institute, Work, Health & Performance, Radboud University, Nijmegen, The Netherlands; i Department of Public and Occupational Health, Amsterdam University Medical Center (UMC), Vrije Universiteit, Amsterdam, The Netherlands; j Amsterdam Public Health Research Institute, Societal Participation and Health, Amsterdam, The Netherlands

**Keywords:** Leadership, occupational health, staff development, career mobility, employment, universities

## Abstract

**BACKGROUND:**

In the changing world of work, there is an urgency to gain insight into determinants of the employability among support staff workers with long tenure whose functions may become outdated as their competencies may no longer match the requirements of future jobs.

**OBJECTIVE:**

The specific aim of this study was to investigate the relationship between transformational leadership and employability.

**METHODS:**

Support staff (*n* = 236) from a university participated in an online questionnaire focusing on five dimensions of employability (occupational expertise, anticipation and optimization, personal flexibility, corporate sense, and balance) and transformational leadership (identifying and articulating a vision, providing an appropriate model, fostering the acceptance of group goals, providing individual support, and intellectual stimulation.

**RESULTS:**

Identifying and articulating a vision (β= 0.247, *p* < 0.001), providing an appropriate model (β= 0.196, *p* = 0.002), fostering the acceptance of group goals (β= 0.298, *p* < 0.001) and providing individual support (β= 0.258, *p* < 0.001) were associated with higher balance scores. No significant associations were found between the transformational leadership subscales and the other dimensions of employability.

**CONCLUSION:**

The current study found that just one specific dimension of transformational leadership was associated with only one aspect of employability for our target group of long-term employed support staff workers with a high level of job security.

## 1 Introduction

Under proper work conditions, being employed has a positive effect on humans. Unemployment is related to less physical and psychological well-being [1, 2] as well as to reduced happiness [3]. Therefore, it is crucial to sustain one’s ability to work at least until one’s retirement age. This is increasingly becoming a challenge as, nowadays, the labor market is characterized by an increasing job insecurity causing an even greater importance to focus on enhancing one’s employability (i.e., career potential) in order to get and maintain a job [4, 5]. Employability can be defined as “the continuous fulfilling, acquiring or creating of work through the optimal use of competences” [6].

Despite the generally increasing job insecurity, there still is a considerable group of employees, albeit rapidly reducing in numbers, that experiences job security, i.e., permanent employment contracts and long tenures. Yet, if someone works for many years in the same function within one particular organization only, this person might get less opportunities to continuously develop the broader range of competencies that the current external labor market asks for [5, 7]. Specifically, due to digitalization and rapid technological progress, various functions are changing or might even get outdated [8] at an ever-increasing rate. As a result, this group of employees that work for many years in the same function within one particular organization only is at risk as their competencies may no longer fit current vacancies, herewith endangering their sustainable employability.

We argue that this vulnerable group of workers may benefit from transformational leadership [9]. Transformational leadership is characterized by inspirational leaders who stimulate their subordinates to share and commit to the leaders’ vision by modifying their attitudes, values and beliefs [10, 11]. Transformational leaders are strongly involved in their subordinates’ development [12] and strive to exploit their potential and to develop their capabilities [13]. They stimulate them intellectually [11] and motivate them to “perform beyond the level of expectations” [14]. Next to this, transformational leaders behave as role models who act in line with their values [11]. Altogether, we posit that establishing a transformational leadership style could benefit our target group’s employability, as it is associated with facilitating learning and development [15, 16], which are key to gain knowledge and to develop skills and competencies [17–19].

Research on associations between transformational leadership and employability is sparse. In previous scholarly work in this field, Camps and Rodríguez already found transformational leadership to be positively related to employability [20]. However, their study only included academic university staff participants. Considering that academics often have termed contracts, and thus experience less job security, they are usually quite focused on actively improving their employability [21]. Our target group experiencing a high level of job security, and having permanent work contracts and long tenures is relevantly different from the sample in the previous study, and it might be that the lack of attention although there is less urgency to protect and further enhance their employability for the external labor market [21] there is a need for more scholarly work to better understand the association under interest for our target group. After all, in the light of the need to protect all categories of workers’ employability over time [4], we posit that investigating the predictive validity of transformational leadership is important. Particularly, we do not know whether the findings of Camps and Rodríguez [20] dealing with a vulnerable group of academics can be generalized to a group of workers with more job security and long tenure. The aim of this study was therefore to investigate the relationship between transformational leadership and employability using a sample of Dutch non-academic support staff which is characterized by permanent work contracts and long tenure.

## 2 Method

### 2.1 Procedure and sample

A cross-sectional research was conducted to examine the relationship between transformational leadership and employability. For this purpose, an online questionnaire was sent to all employees of the central support staff division of a Dutch university. We sent an email to the employees explaining that we intended to conduct research as part of a project to improve the university’s policy regarding sustainable employability. For this matter, the employees were asked to participate in the questionnaire which could be reached by an integrated link for 23 days. After 18 days they received a reminder in the official monthly newsletter of the university’s support staff division.

The online questionnaire was sent to all 1,234 employees of the university’s support staff division of which 309 employees (25,04%) gave consent to participate by completing at least part of the questionnaire. We included data of 236 participants (76,38%) who fully completed the questionnaire.

### 2.2 Dependent variable: Employability

We used the thoroughly validated 22-item short version [22] of the original scale developed by Van der Heijde and Van der Heijden [23]. The instrument included five dimensions: occupational expertise defined as domain-related knowledge and skills (five items), anticipation and optimization defined as preparing for and adapting to future changes in a personal and creative manner, and striving for the best possible results (four items), personal flexibility defined as the capacity to adapt easily to all kinds of changes in the internal and external labor market that do not pertain to one’s immediate job domain (five items), corporate sense defined as the participation and performance in different work groups, such as, organizations, teams, occupational communities and other networks, herein sharing responsibilities, knowledge, experiences, feelings, credits, failures, goals, etc. (four items), and balance defined as compromising between opposing employers’ interests as well as one’s own opposing work, career, and private interests (employee) and between employers’ and employees’ interests (four items). All items were scored on six-point Likert scales. Occupational expertise was measured using five items (sample item: “During the past year, I was, in general, competent to perform my work accurately and with few mistakes”; answering categories from “not at all” to “extremely”). Anticipation and optimization was measured using four items (sample item: “During the past year, I associated myself with the latest developments in my job domain”; answering categories from “never” to “very often”). Personal flexibility was measured using five items (sample item: “How easily would you say you can adapt to changes in your workplace?”; answering categories from “very badly” to “very well”). Corporate sense was measured using four items (sample item: “In my organization, I take part in forming a common vision of values and goals”; answering categories from “never” to “very often”). Balance was measured using four items (sample item: “The time I spend on my work and career development on the one hand and my personal development and relaxation on the other are evenly balanced”; answering categories from “not at all” to “to a high degree”). The responses were given on different six-point Likert scales according to the respective statements (for example “1 = ‘very little’ or ‘very low”’; “2 = ‘relatively little’ or ‘rather low”’; “3 = ‘not much’ or ‘not very high”’; “4 = ‘a fair amount’ or ‘reasonably high”’; “5 = ‘a great deal’ or ‘high”’; “6 = ‘a very great deal’ or ‘very high”’, respectively). A higher mean score indicated a higher degree of employability.

### 2.3 Independent variables: Transformational leadership

We used 20 items of the transformational leadership inventory [11] that were equally distributed over five dimensions: identifying and articulating a vision defined as behavior of the leader characterized by finding new opportunities for their team or organization, and developing and communicating a vision of the future that inspires their employees (sample item: “Inspires others with their plans for the future.”); providing an appropriate model defined as the leader setting an example by acting in line with their values (sample item: “Leads by example.”); fostering the acceptance of group goals defined as the leader’s behavior that stimulates the employees to work cooperatively toward a shared goal (sample item: “Gets the group to work together for the same goal.”); providing individual support defined as behavior of the leader that is characterized by respect for their employees and concern for their personal needs and feelings (sample item: “Shows respect for my personal feelings.”), and intellectual stimulation defined as the leader motivating their employees to question their assumptions regarding their work and rethink their way of working (sample item: “Challenges me to think about old problems in new ways.”). The responses were given on a seven-point Likert scale ranging from “strongly disagree” to “strongly agree” whereby a higher mean score indicated a higher degree of transformational leadership. The subscales for transformational leadership were translated from English into Dutch by a team of professional translators and the authors of this article, using the translation back-translation methodology [24].

### 2.4 Potential confounders

We included questions regarding gender (male/female/other), age (in years), level of education (primary school/middle school/high school/vocational school/Bachelor’s degree; Master’s degree; PhD), tenure (<5 years/5–10 years/11–20 years/>21 years), contract type (termed/permanent) and whether they supervise other employees (yes/no) and contract hours (hours per week).

### 2.5 Data analyses

Firstly, we conducted descriptive analyses for the dependent and independent variables as well as for the potential confounders. Next, statistical assumptions were checked and the following analyses were repeated with bootstrapping due to violation of the assumption of homoscedasticity of variance.

Subsequently, we performed hierarchical regression analyses. In Step 1, we tested the predictive value of each of the independent variables (the five dimensions of transformational leadership) in separate models. In Step 2, we added the potential confounders to the model. Confounding was considered relevant if the standardized regression weights of the independent variables changed at least with 10% after adding them to the model.

As there were five dependent variables (the five dimensions of employability: ‘occupational expertise’, ‘anticipation and optimization’, ‘personal flexibility’, ‘corporate sense’ and ‘balance’), a significance level of.01 rather than.05 was chosen to reduce the chance of multiple testing errors. Analyses were performed using SPSS 23.

## 3 Results

### 3.1 Sample

Our sample consisted of 134 women, 100 men, and two other. Age ranged from 20 to 65 years. Educational level was distributed as follows: high school (7.6%), vocational education (41.9%), Bachelor’s/Master’s degree; PhD (50.8%). Two-third of the sample had been employed at the university for more than 5 years. Eighty-six percent of the employees had a permanent employment contract and 19 percent had supervisory function. On average, the employees worked 32.4 hours per week (Table 1).

**Table 1:** Sample characteristics (*n* = 236) and descriptives of transformational leadership and employability

Demographic characteristics	Mean	SD	*n* (%)
Female gender (*n*/%)			134 (56.8)
Age (years)	47.4	11.0
Education: Vocational level or lower			117 (49.6)
Work characteristics
Permanent contract			204 (86.4)
Amount of work hours	32.4	6.5
Supervisory role			46 (19.1)
Tenure
<5 years			77 (32.6)
> = 5 and <10 years			36 (15.3)
> = 10 and <20 years			70 (29.7)
> = 20 years			53 (22.5)
Transformational leadership
Vision	4.6	1.4
Role model	4.5	1.4
Group goals	4.9	1.3
Individual support	5.4	1.4
Intellectual stimulation	4.5	1.3
Employability
Occupational expertise	4.6	0.6
Anticipation and optimization	3.8	0.9
Personal flexibility	4.6	0.6
Corporate sense	4.4	0.8
Balance	4.2	0.7

### 3.2 Associations between transformational leadership and employability

With regards to transformational leadership, the participants scored the highest on the dimension ‘providing individual support’ (M = 5.38, SD = 1.36) and lowest on ‘intellectual stimulation’ (M = 4.47, SD = 1.30) (Table 1). Regarding employability, participants scored the highest on the dimension ‘occupational expertise’ (M = 4.64, SD = 0.58) and lowest on ‘anticipation and optimization’ (M = 3.76, SD = 0.88) (Table 1).

No significant associations were found between the transformational leadership subscales and the employability dimensions of ‘occupational expertise’, ‘anticipation and optimization’, ‘personal flexibility’ and ‘corporate sense’ (Table 2). Yet, we found significant associations between four transformational leadership variables and the employability dimension ‘balance’ (Table 2). In the separate models, higher scores for ‘identifying and articulating a vision’ (β= 0.247, *p* < 0.001), ‘providing an appropriate model’ (β= 0.196, *p* = 0.002), ‘fostering the acceptance of group goals’ (β= 0.298, *p* < 0.001) and ‘providing individual support’ (β= 0.258, *p* < 0.001), respectively, were related to a higher ‘balance’ score. Adding the control variables to the models did not change the models relevantly. The ‘intellectual stimulation’ subscale of transformational leadership was not significantly associated with the employability dimension ‘balance’.

**Table 2:** Regression models for associations of transformational leadership (independent variables) with employability (dependent variables) (*n* = 236)

Employability		Occupational			Anticipation and			Personal flexibility			Corporate sense			Balance		
		expertise			optimization											
Transformational		β	99% CI		β	99% CI		β	99% CI		β	99% CI		β	99% CI	
leadership			Lower	Upper		Lower	Upper		Lower	Upper		Lower	Upper		Lower	Upper
Vision	CM	–0.087	–0.110	0.035	–0.034	–0.133	0.089	–0.006	–0.081	0.076	–0.087	–0.150	0.048	0.247	0.042	0.213
	AM	–0.081	–0.108	0.038	–0.033	–0.133	0.090	0.009	–0.074	0.082	–0.057	–0.131	0.065	0.234	0.035	0.207
Role model	CM	–0.115	–0.121	0.023	–0.096	–0.171	0.047	0.013	–0.071	0.083	–0.068	–0.138	0.059	0.196	0.015	0.185
	AM	–0.107	–0.118	0.027	–0.106	–0.178	0.041	0.021	–0.068	0.086	–0.048	–0.124	0.069	0.185	0.009	0.180
Group goals	CM	–0.019	–0.082	0.066	–0.043	–0.142	0.084	0.049	–0.057	0.103	0.007	–0.097	0.106	0.298	0.072	0.242
	AM	–0.006	–0.077	0.072	–0.038	–0.138	0.087	0.068	–0.047	0.111	0.040	–0.075	0.123	0.294	0.069	0.240
Individual support	CM	–0.001	–0.073	0.072	–0.061	–0.150	0.070	0.072	–0.045	0.110	–0.067	–0.138	0.060	0.258	0.048	0.217
	AM	0.008	–0.069	0.076	–0.058	–0.147	0.072	0.086	–0.037	0.116	–0.042	–0.121	0.071	0.252	0.045	0.213
Intellectual stimulation	CM	–0.138†	–0.137	0.013	–0.013	–0.125	0.106	0.005	–0.079	0.084	–0.006	–0.107	0.100	0.167	0.000	0.180
	AM	–0.122	–0.131	0.022	–0.005	–0.120	0.113	0.033	–0.066	0.097	0.043	–0.076	0.129	0.162	–0.004	0.178

CM: Crude Model; AM: Adjusted Model, corrected for contract type (termed/permanent), supervisory function (yes/no) and number of work hours.

## 4 Discussion

### 4.1 Reflection on the outcomes

The current study examined the relationship between transformational leadership and employability in academic support staff with long tenures. Contrary to our expectations, transformational leadership was not found to be positively related to employability for four of the five dimensions of employability. We only found one significant association, with a small effect size, with transformational leadership for the employability dimension ‘balance’. Previous research showed mixed findings regarding transformational leadership and employability in general, with larger effect sizes overall. Some studies found a positive [20, 25, 26], another study did only find an indirect relationship between transformational leadership employability, through work-related flow [27]. Considering the relatively scarce amount of studies that examined the focal relationship in our scholarly work, it might be possible that its pattern is not as straight-forward as assumed in our specific target group.

Other research in this domain has also found mixed findings for the relationship between transformational leadership and some other factors that are important in the light of an individual’s employability, such as innovative behavior and creativity [23]. Transformational leadership is often assumed to enhance an employee’s innovative behavior [28] and creativity [29]. In the university context, these factors are key to the scientists, but not necessarily for the support staff, which might explain our non-significant findings.

Yet, there are also studies that found a negative [30, 31] or no relationship [32, 33]. Another study that is also interesting in this regard comprises the work by Eisenbeiß and Boerner [34] who reported that followers of transformational leaders can develop a dependence on their leader which, in turn, reduces their creativity. As such, transformational leadership can be a double-edged sword as this negative indirect effect attenuates the positive influence of transformational leadership on followers’ creativity.

Perhaps there is a similar effect regarding employability: although transformational leaders promote their employees’ development by stimulating exploiting their own potential [13], transformational leaders also make their followers committed to their visions and stimulate them to align with their attitudes [10]. Yet, if leaders focus on another subject rather than on their subordinates’ employability, the attention of the employee for actively managing their employability might be reduced. The latter might undermine the positive impact of transformational leadership on employability.

Moreover, considering that our target group, being support staff in a university setting, experiences a high level of job security and long tenures, there might be a leadership culture that values the balance dimension more in comparison with the other employability dimensions. This could explain that we found a significant association between transformations leadership and the employability dimension ‘balance’ but not for the other four dimensions.

### 4.2 Methodological considerations

The focus in our research on academic support staff may have raised a generalization problem, as our findings might not be applicable to other labor groups (for example physical work instead of office work). However, since our main interest was the relationship between transformational leadership and employability, and as we did not find any relevant confounding effects due to gender, level of education, or age, we expect that our findings will be generalizable to other populations of workers with different demographic characteristics. Moreover, our sample was heterogeneous with regards to educational level as it stands out that more than half of the participants had a university degree. It might be possible that there are academics who dropped out from an academic career but still prefer to work in an organization that is characterized by an academic culture. In the Netherlands, tenure for academics is difficult to obtain in the field of research, while a job as a support staff member offers greater stability and job security. For the present research question, the inclusion of participants with lower as well as higher educational levels in our sample offers a strong basis for testing associations. With regards to representativeness, given that our primary interest was the association between transformational leadership and employability, the heterogeneity of our sample is a strength, and outweighs the low response rate. It should be noted that 32% had a tenure for less than 5 years, which does not qualify for long tenure. However, we did not only aim to include workers with a minimum number of tenured years as we aimed to include this group of which we know they generally are characterized as having long tenures. The group with shorter tenure is mainly a group with a lower age, that is very likely to be at the start of a longer tenure.

Given the cross-sectional design of our study, it is not possible to conclude safely about causal relationships between the independent and dependent variables [35]. Next, as the independent and dependent variables are all measured by means of self-reports in a survey approach, response set consistencies might have occurred. The participants’ answers could also have been impacted by a social desirability bias [36]. We have tried to reduce this risk by assuring anonymity and communicating to the participants “that there are no right or wrong answers” [37]. In addition, the scale endpoints differed for the independent and dependent variables which is also a strategy that is used to reduce this bias [38].

Although we tested possible confounding effects due to demographics and some work characteristics, possible confounding effects caused by other individual (e.g., health status) or work characteristics (e.g., work load, autonomy) cannot be ruled out.

### 4.3 Implications

The results from the current study contribute to a greater understanding about how employees can be facilitated in staying employable and meeting the labor market’s demands. Yet, the current study found that just one specific dimension of transformational leadership was associated with only one aspect of employability for our target group of long-term employed workers with a high level of job security. This outcome could implicate that a transformational leadership style is not effective in promoting the employability of this specific target group. Considering the mixed findings regarding the association between transformational leadership and employability in general [20, 25, 26], future research could investigate whether this relationship is moderated by other factors, for example the focus of the leader’s vision and values. As the employee’s career development depends on values, approach and skills from their manager, it is crucial to make sure that organizations take care of the development of sound leadership competencies wherein leaders are instructed to stimulate their employees to take responsibility for their own employability, but at the same time, encourage and stimulate their subordinates’ career development themselves, in their role as leaders, as well. In order to make sure that the employees’ career sustainability [39] is protected and ideally enhanced throughout the career span, organizations with long-term employed workers with a high level of job security need to be aware of the fact that, regular career conversations and guidance on their sustainable employability is of utmost importance, over and above appraisals of their current performance.

This means that attention for employability enhancement should be part of the leaders’ vision, values and education and that they know how to translated (operationalize) these in concrete HR practices. In light of this, it may be interesting to use the Job Demands-Resources (JD-R) framework [40] that has proven to be applicable to many occupational and organizational settings as a guiding framework for managers that take care of their employees’ current functioning as well as their employability over time.

To enhance workers’ employability, attention should be directed towards multiple facets in practice. Key recommendations include investing in employability growth, enhancing communication, and giving precedence to leadership development. For instance, further investments can be made in the employability growth of experienced employees through mentorship programs, continuous education and upskilling, and fostering a climate wherein learning and development are prioritized. Communication can be improved by ensuring clear information regarding necessary changes, involving employees in decision-making processes, and valuing their ideas and input. Leadership development should take precedence through the training and guidance of leaders aimed at fostering transformational leadership, improving their ability to inspire employees and instill an awareness of the significance of inspirational leadership, individual attention, intellectual stimulation, and clear expectations [11]. This enables organizations to harness the valuable contributions of their experienced employees and build a resilient workforce for the future.

## Ethical approval

According to Dutch law, given that the questionnaire was anonymous, this study was exempt from Medical Ethical Review.

## Informed consent

Employees gave consent by completing at least part of the questionnaire.

## Conflict of interest

None reported.

## Data Availability

Anonymous data were collected for the aim of this study; they are available upon request from the corresponding author.
